# Long non-coding RNA XIST promotes retinoblastoma cell proliferation, migration, and invasion by modulating microRNA-191-5p/brain derived neurotrophic factor

**DOI:** 10.1080/21655979.2021.1918991

**Published:** 2021-05-04

**Authors:** Yifan Xu, Zheng Fu, Xuexia Gao, Ruifeng Wang, Qiuming Li

**Affiliations:** a Department of ophtalmology, The First Affiliated Hospital of Zhengzhou University, Zhengzhou, Henan, China; b Department of ophtalmology, Zhengzhou Second Hospital, Zhengzhou, Henan, China

**Keywords:** XIST, retinoblastoma, miR-191-5p, bdnf

## Abstract

Long non-coding RNA (lncRNA) X–inactive specific transcript (XIST) is oncogenic in multiple cancers. Herein, the present study is aimed at delving into how XIST functions in retinoblastoma (RB) and investigating its underlying mechanism. In this study, XIST, miR-191-5p, BDNF mRNA, and BDNF expression levels in RB tissues or cell lines were examined by quantitative real-time polymerase chain reaction (qRT-PCR) or Western blot. The models of gain-of-function and loss-of-function were established by the transfection of pcDNA3.1-XIST, XIST siRNA, and miR-191-5p mimics and inhibitors into SO-Rb50 and Y79 cells, respectively. RB cell proliferation, migration, invasion, and apoptosis were detected employing cell counting kit-8 (CCK-8), Transwell, and terminal deoxynucleotidyl transferased UTP nick end labeling (TUNEL) assays. The regulatory relationships among XIST, miR-191-5p, and BDNF were affirmed utilizing bioinformatics analysis, luciferase reporter assay, qRT-PCR, as well as Western blot. We reported that, XIST expression was markedly elevated in RB tissue and RB cells. XIST overexpression accelerated RB cell proliferation, migration, and invasion, and attenuated RB cell apoptosis but miR-191-5p exerted the opposite effects. Besides, BDNF expression was inhibited by miR-191-5p in both mRNA and protein levels. XIST indirectly improved BDNF expression by repressing miR-191-5p expression as a competitive endogenous RNA. In conclusion, XIST expression is abnormally elevated in RB tissues and XIST can modulate proliferation, migration, invasion, and apoptosis of RB cells by regulating miR-191-5p/BDNF axis.

## Graphical abstract

**Figure:**
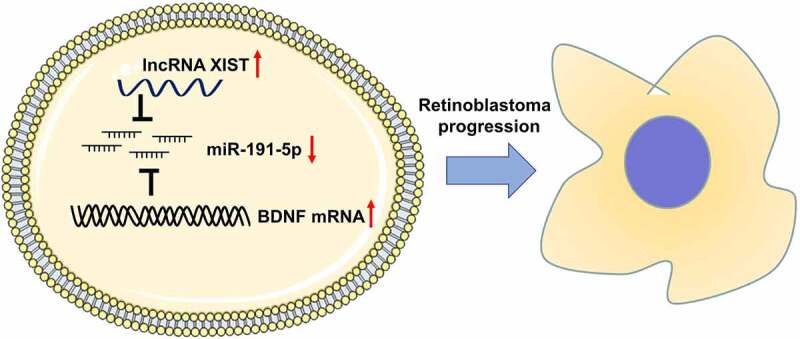

## 1. Introduction

Retinoblastoma (RB) is a common malignancy among children [1–3]. Therapeutic strategies for RB include enucleation, chemoreduction, and local treatments, such as laser, freezing, and radiotherapy [4]. However, the therapeutic effect and prognosis of RB are still unsatisfying [5]. Hence, it is vital to delve into the molecular mechanism underlying RB tumorigenesis and development to unmask novel targets for RB diagnosis and treatment.

Currently, increasing studies substantiate the importance of lncRNA in tumorigenesis and tumor development [6]. Long non-coding RNA (lncRNA) X–inactive specific transcript (XIST) figures prominently in X chromosome inactivation [7]. Specifically, XIST serves as a tumor-promoting lncRNA or tumor suppressor in various human malignancies [7]. Besides, XIST expression is elevated in RB tissues and cell lines, and after knocking down XIST, the proliferation of RB cells is restrained and apoptosis is induced [8]. XIST increases the expression levels of ZEB1 and ZEB2 as a competitive endogenous RNA (ceRNA) of miR-101, facilitating epithelial-to-mesenchymal transformation of RB cells and RB progression [9]. To date, the previous studies imply that XIST may be a cancer-promoting factor in RB, yet the role and molecular mechanism of XIST in the tumorigenesis and development of RB have not been fully elucidated.

MicroRNA (miRNA), defined as a small and short non-coding RNA, usually modulates gene expression through binding with the 3ʹ- untranslated region (3ʹ-UTR) of target mRNA, thereby partaking in regulating cell proliferation, differentiation, as well as apoptosis [10]. Previous studies show that various miRNAs are involved in the tumorigenesis and development of RB [11–13]. MiR-191-5p possesses multiple biological effects in cancer biology. For instance, in osteosarcoma, miR-191-5p is highly expressed, which is associated with the unfavorable prognosis of patients [13]. In renal cell cancer, miR-191-5p impedes cancer cell proliferation, migration, and invasion [14]. Nonetheless, the specific function and mechanism of miR-192-5p in RB warrants further investigation.

Brain derived neurotrophic factor (BDNF) features prominently in the survival, differentiation, growth, and development of neurons [15,16]. In cancer biology, BDNF expression is increased in bladder cancer, glioma, gastric cancer, colorectal cancer, breast cancer, and other human malignancies [17]. Moreover, BDNF also participates in RB progression. Highly expressed BDNF, by activating PI3K/AKT signaling pathway, boosts the proliferation and invasion of RB cells [16]. Notwithstanding the aforementioned reports, the upstream regulatory mechanism of BDNF in RB progression has not been completely clarified.

This study further substantiated the roles of XIST, miR-191-5p, and BDNF in RB progression and clarified their mutual regulation mechanisms. We hypothesized that XIST expression was increased in RB tissues and XIST could facilitate cell proliferation, migration, and invasion while inhibiting apoptosis by regulating miR-191-5p/BDNF in RB.

## 2. Materials and methods

### 2.1 Clinical samples

Human tissues were derived from 34 RB patients in the First Affiliated Hospital of Zhengzhou University from August 2013 to August 2018. The study was approved by the Ethics Committee of the First Affiliated Hospital of Zhengzhou University (Approval No. 201,301,008). The guardians of patients signed the consent form before the tissue sample collection.

### 2.2 Cell culture and transfection

Human RB cell lines (HXO-RB44, Y79, WERI-Rb-1, and SO-RB50), together with normal human retinal pigment epithelial cell ARPE-19, were available from the Cell Center of Chinese Academy of Sciences (Shanghai, China). Then, the above cells were cultured in Dulbecco Modified Eagle Medium (DMEM, Life Technologies, Grand Island, NY, USA) with 10% fetal bovine serum (FBS, HyClone, Little Chalfont, UK), 100 U/mL penicillin, and 100 μg/mL streptomycin (Hyclone, Logan, UT, USA), and cultured at 37°C with 5% CO_2_. Besides, the medium was replaced at the interval of every 2–3 d. pcDNA empty vector (NC), pcDNA-XIST, siRNA negative control (si-NC), siRNA against XIST (si-XIST), miRNA control (miR-NC), miR-191-5p mimics, as well as miR-191-5p inhibitors were obtained from GenePharma Co., Ltd. (Shanghai, China), and the transfection was performed with Lipofectamine®3000 (Invitrogen, Carlsbad, CA, USA) after the cell culture in serum-free medium for 12 h. After 24 h, the medium was replaced by complete medium, and the cells were collected for 48 h. Then the cells were harvested for examining the transfection efficiency.

### 2.3 Quantitative real-time polymerase chain reaction (qRT-PCR)

After TRIzol reagent (Invitrogen, Carlsbad, CA, USA) was utilized to extract the total RNA from cells or RB tissues, and the purity and concentration of total RNA were examined, reverse transcription into cDNA was carried out with SuperScript First-Stand Synthesis System (TaKaRa, Dalian, China) in accordance with the manufacturer’s instructions, followed by the amplification detection with SYBR Premix Ex Taq II (TaKaRa, Dalian, China) on RT-PCR system (StepOneTM, Applied Biosystems, Darmstadt, Germany). The primers were designed and synthesized by Sangon Co., Ltd. (Shanghai, China) (Table 1).

**Table 1.** The PCR primer sequence used in this study

Name	Primer sequences
XIST	Forward: 5-GCTCTTCATTGTTCCTATCTGCC-3′
	Reverse: 5-TGT GTAAGTAAGTCGATAGGAGT-3′
miR-191-5p	Forward: 5′-CGGAATCCCAAAAGCAGCTG-3′
	Reverse: 5′-TGTCGTGGAGTCGGCAATTG-3′
GAPDH BDNF	Forward: 5′-TGATGACATCAAGAAGGTGG-3′
	Reverse: 5′-TTACTCCTTGGAGGCCATGT-3′ Forward: 5′-TTATTTCATACTTCGGTTGC-3′ Reverse: 5′-ATGGGATTACACATTGGTCTC-3′
U6	Forward:5′-CTCGCTTCGGCAGCACA-3′
	Reverse: 5′-ACGCTTCACGAATTTGCGT-3′

### 2.4 Western blot

Protein extraction kit (Beyotime, Shanghai, China) was employed to extract total proteins from RB cells, and BCA method was utilized to determine the protein concentration of the samples. After the denaturation, the separation of 20 μg of total protein was conducted by SDS-PAGE and then the protein was transferred to PVDF membranes (PVDF, Millipore, Billerica, MA, USA). After the membranes were blocked by 5% skimmed milk for 1 h at ambient temperature, the membranes were incubated with primary antibody (Anti-BDNF antibody, mouse anti-human monoclonal antibody, ab203573, Abcam, Shanghai, China, 1:1000) at 4°C overnight. After that, the membranes were rinsed with TBST, and then incubated with horseradish peroxidase labeled secondary antibody (Goat Anti-Mouse IgG, ab205719, Abcam, Shanghai, China, 1:2000 dilution) at ambient temperature for 1 h. After rinsing the membranes with TBST, the electrochemical luminescence (ECL) kit (Tanon, Shanghai, China) was employed to develop the protein bands.

### 2.5 Cell counting kit-8 (CCK-8) assay

The cells were harvested to prepare a single-cell suspension and the density was modulated subsequent to cell counting. 1000 cells were transferred into each well of a 96-well plate. On the following day, 10 μL of CCK-8 solution (Beyotime, Shanghai, China) was added into each well, and blank control wells (only containing the medium and CCK-8 solution) were established. After 2 h, a microplate reader was employed to quantify the absorbance (OD) value of each well at 450 nm wavelength. With the same method, the OD values of the wells were detected every 24 h for 4 d.

### 2.6 Transwell assay

Transwell experiments were employed to detect RB cell migration and invasion. After being dispersed with 0.25% trypsin, RB cells were centrifuged and resuspended with serum-free medium. In the invasion experiment, the Transwell chambers (pore size: 8 µM, Corning, Beijing, China) were coated with a layer of Matrigel; Matrigel was not utilized in the migration experiment. Subsequently, the transfected cells were inoculated in the upper compartment of each Transwell chamber (5 × 10^4^ cells/well), with the lower compartment added with the medium containing 10% FBS. After the culture at 37°C for 24 h, cells failing to migrate or invade were removed from the upper surface of the filter. After being fixed with 4% paraformaldehyde for 10 min and, subsequently, stained with 0.5% crystal violet, the cells on the below surface of the filter were rinsed with tap water, followed by being dried and counted under an inverted microscope.

### 2.7 Dual-luciferase reporter assay

Wild type (MUT) and mutant type (Mut) luciferase reporter plasmids pmiR-GLO-WT/Mut XIST and miR-191-5p mimics/miRNA NC were co-transfected into RB cells. 48 h later, the dual-luciferase reporter assay system (Promega, Madison, WI, USA) was employed to quantify the luciferase activity. The targeting relationship between miR-191-5p and the 3ʹUTR of BDNF was verified using the same method.

### 2.8 The terminal deoxynucleotidyl transferased UTP nick end labeling (TUNEL) assay

TUNEL assay kit (Beyotime, Shanghai, China) was used to evaluate the apoptosis of RB cells. In brief, the medium was discarded and PBS was employed to rinse the RB cells. After being fixed with immunostaining fixative for 30 min, the cells were rinsed once with PBS. Subsequently, the cells were incubated with immunostaining washing solution in the ice bath for 2 min, followed by the incubation with TUNEL test solution at 37°C in the dark for 60 min, and then the cells were rinsed with PBS 3 times. Then, the samples were sealed with anti-fluorescence quenching sealing liquid and observed under a fluorescence microscope.

### 2.9 Statistical analysis methods

The statistical analysis was conducted employing SPSS22.0 statistical software (SPSS Inc., Chicago, IL, USA). Measurement data were shown as ‘mean ± standard deviation (x ± s)’. The independent sample *t*-test was employed to compare the differences between the two groups. A Chi-square test was employed for the comparison of inter-group rate and composition ratio. *p* < 0.05 indicated that the difference was of statistical significance.

## 3. Results

The study was aimed at elucidating the role of XIST in regulating the proliferation, migration, invasion, and apoptosis of RB cells, and exploring the regulatory relationship among XIST, miR-191-5p, and BDNF. We hypothesized that XIST expression was increased in RB tissues and XIST could facilitate RB cell proliferation, migration, and invasion, and inhibit cell apoptosis by regulating miR-191-5p/BDNF axis. RB cell lines SO-Rb50 and Y79 were transfected with XIST overexpressed plasmids, XIST siRNA, miR-191-5p mimics, or miR-191-5p inhibitor. The expressions of XIST, miR-191-5p, and BDNF were determined using qRT-PCR or Western blot. The binding relationships between XIST and miR-191-5p, as well as miR-191-5p and BDNF were investigated by dual-luciferase reporter gene assay. Functional experiments, including CCK-8, Transwell, and TUNEL assays, were performed to detect RB cell growth, migration, invasion, and apoptosis.

### 3.1 XIST expression was elevated in RB tissues, and its high expression was related to unfavorable pathological characteristics

**Figure 1.**
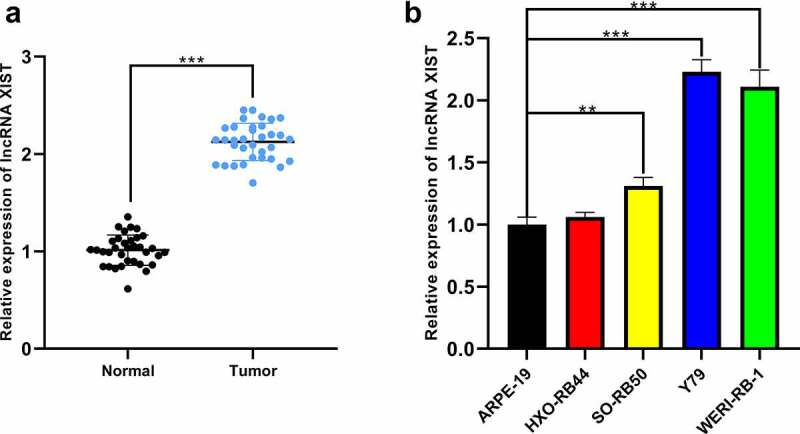
**The expression of XIST in RB tissues and cell lines**. (a) The expression of XIST in normal and RB tissues was detected by qRT-PCR. (b) The expression of XIST in RB cell lines and normal human retinal pigment epithelial cells was detected by qRT-PCR

XIST expression in RB tissues was quantified by qRT-PCR, and it was elevated remarkably in RB tissues (Figure 1(a)). Additionally, XIST expression in RB cells SO-Rb50, Y79, and WERI-Rb-1 was markedly higher than that in normal human retinal pigment epithelial cell line ARPE-19 (Figure 1(b)). We then analyzed the correlation between XIST expression and pathological characteristics of RB patients through Chi-square test, and the data implied that there was no significant correlation between XIST expression and gender or age of the patients, whereas significant correlation was observed between XIST expression and tumor size, choroidal nerve invasion, optic nerve invasion, and tumor staging, indicating that XIST partook in the progression of RB (Table 2).

**Table 2.** Chi-square test of RB patient characteristics and XIST expression

Characteristics	Number of cases	Relative expression of XIST		Chi-square	P value
		High	Low		
Total cases	34	22	12		
Gender				0.875	0.476
Male	19	11	8		
Female	15	11	4		
Age(years)				0.172	0.738
≤3	18	12	6		
>3	16	10	6		
Tumor size(mm)				7.993	0.008
≤10	12	4	8		
>10	22	18	4		
Choroidal nerve invasion				4.310	0.045
Positive	22	17	5		
Negative	12	5	7		
Optic nerve invasion				4.975	0.031
Positive	20	16	4		
Negative	14	6	8		
Stages				5.720	0.023
Group A-C	23	18	5		
Group D-E	11	4	7

** denotes *p* < 0.01 and *** denotes *p* < 0.001

### 3.2 XIST affected the proliferation, migration, invasion, and apoptosis of RB cells

**Figure 2.**
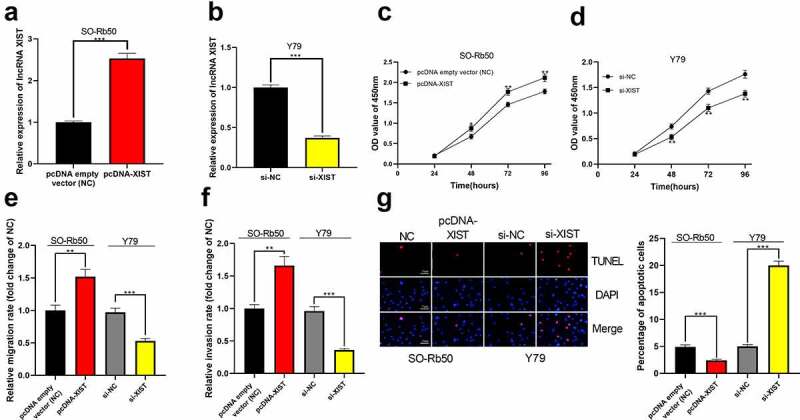
**The biological effects of XIST on the malignant phenotypes of RB cells**. (a-b) Cell models of XIST overexpression and knockdown were established in SO-Rb50 and Y79 cell lines, respectively. (c-d) The effect of XIST overexpression or knockdown on RB cell proliferation was detected by CCK-8 assay. (e-f) The effect of XIST overexpression or knockdown on RB cell migration and invasion was detected through Transwell experiment. (g) The effect of XIST overexpression or knockdown on RB cell apoptosis was assessed through TUNEL experiment

Next, using SO-Rb50 cells and Y79 cells, we established cell models with XIST overexpression and XIST knockdown, respectively (Figure 2(a-b)). Through CCK-8 experiment, it was confirmed that high expression of XIST facilitated the proliferation of SO-Rb50 cells while the knockdown of XIST remarkably impeded the proliferation of Y79 cells (Figure 2(c-d)). Transwell experiments verified that the migration and invasion of cells were observably promoted in XIST overexpression group than in the control group, while the opposite results were observed after XIST was knocked down (Figure 2(e-f)). Additionally, TUNEL experiments indicated that XIST overexpression decreased the apoptosis of SO-Rb50 cells, and XIST knockdown induced the apoptosis of Y79 cells (Figure 2(g)).

* denotes *p* < 0.05, ** denotes *p* < 0.01, and *** denotes *p* < 0.001.

### 3.3 MiR-191-5p was a downstream target of XIST

**Figure 3.**
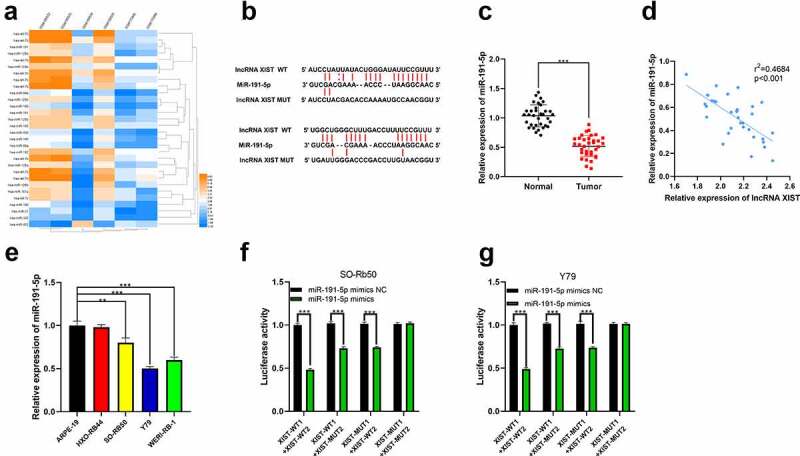
**The interaction between miR-191-5p and XIST**. (a) Multiple miRNAs including miR-191-5p were abnormally expressed in GSE7072. (b) The binding site between XIST and miR-191-5p was predicted by StarBase database. (c) The expression of miR-191-5p in normal tissues and RB tissues was detected by qRT-PCR. (d) The expression of miR-191-5p in RB tissue was negatively correlated with XIST in RB samples. (e) The expression of miR-191-5p in RB cell lines and normal human retinal pigment epithelial cell was detected by qRT-PCR. (f-g) The targeted binding relationship between XIST and miR-191-5p was verified through the dual-luciferase reporter gene assay

For further clarifying the downstream mechanism of XIST, we predicted its downstream miRNA targets through StarBase database. Additionally, we analyzed the miRNAs abnormally expressed in RB tissues with a published miRNA expression profile dataset GSE7072. Interestingly, miR-191-5p was one of the candidate targets of XIST predicted by StarBase, whose expression was markedly decreased in RB tissues compared to normal retinal tissues in GSE7072 (Figure 3(a-b)). Therefore, it was hypothesized that XIST could probably repress miR-191-5p expression by sponging it. Next, miR-191-5p expression in RB tissues was determined by qRT-PCR, and it demonstrated that miR-191-5p was under-expressed in RB tissues (Figure 3(c)). Moreover, XIST and miR-191-5p expression levels were negatively correlated in cancer tissues (Figure 3(d)). MiR-191-5p expression was markedly lower in RB cell lines SO-Rb50, Y79, and WERI-Rb-1 than in normal human retinal pigment epithelial cells (Figure 3(e)). Dual-luciferase reporter assay demonstrated that the luciferase activity of wild type reporter vectors was markedly repressed by miR-191-5p mimics whereas there was no remarkable change in the luciferase activity of reporter vector in XIST-MUT1+ XIST-MUT2 group (Figure 3(f-g)). Based on these data, it could concluded that XIST could target miR-191-5p to suppress its expression.

** denotes *p* < 0.01 and *** denotes *p* < 0.001.

### 3.4 MiR-191-5p affected the proliferation, migration, invasion, and apoptosis of RB cells

**Figure 4.**
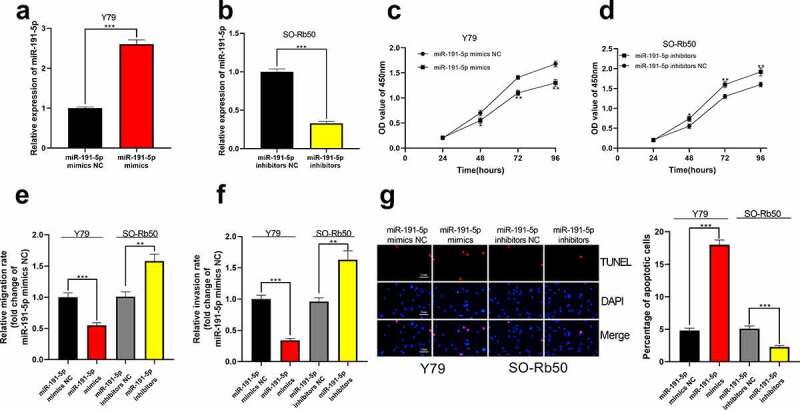
**The effect of miR-191-5p on RB cells**. (a-b) MiR-191-5p overexpression and inhibition cell models were established in Y79 and SO-Rb50 cells, respectively. (c-d) Through CCK-8 assay, the effect of miR-191-5p overexpression or inhibition on RB cell proliferation was detected. (e-f) The effect of miR-191-5p overexpression or inhibition on RB cell migration and invasion was detected by transwell experiment. (g) The effect of miR-191-5p overexpression or inhibition on RB cell apoptosis was detected by TUNEL experiment

Subsequently, cell models with high and low miR-191-5p expression were set up *in vitro* in Y79 and SO-Rb50 cells, respectively (Figure 4(a-b)). Through CCK-8 experiment, it was confirmed that the highly expressed miR-191-5p restrained the proliferation of RB cells, while the proliferation of SO-Rb50 cells transfected with miR-191-5p inhibitor was markedly enhanced (Figure 4(c-d)). Transwell experiment manifested that the migration and invasion of Y79 cells overexpressing miR-191-5p were remarkably lower than those in the control group but the inhibition of miR-191-5p expression dramatically expedited the migration and invasion of SO-Rb50 cells (Figure 4(e-f)). Additionally, it was observed through TUNEL experiment that high miR-191-5p expression facilitated RB cell apoptosis whereas the opposite phenomenon was observed after miR-191-5p expression was inhibited (Figure 4(g)).

* denotes *p* < 0.05, ** denotes *p* < 0.01, and *** denotes *p* < 0.001.

### 3.5 BDNF was the downstream target of miR-191-5p

**Figure 5.**
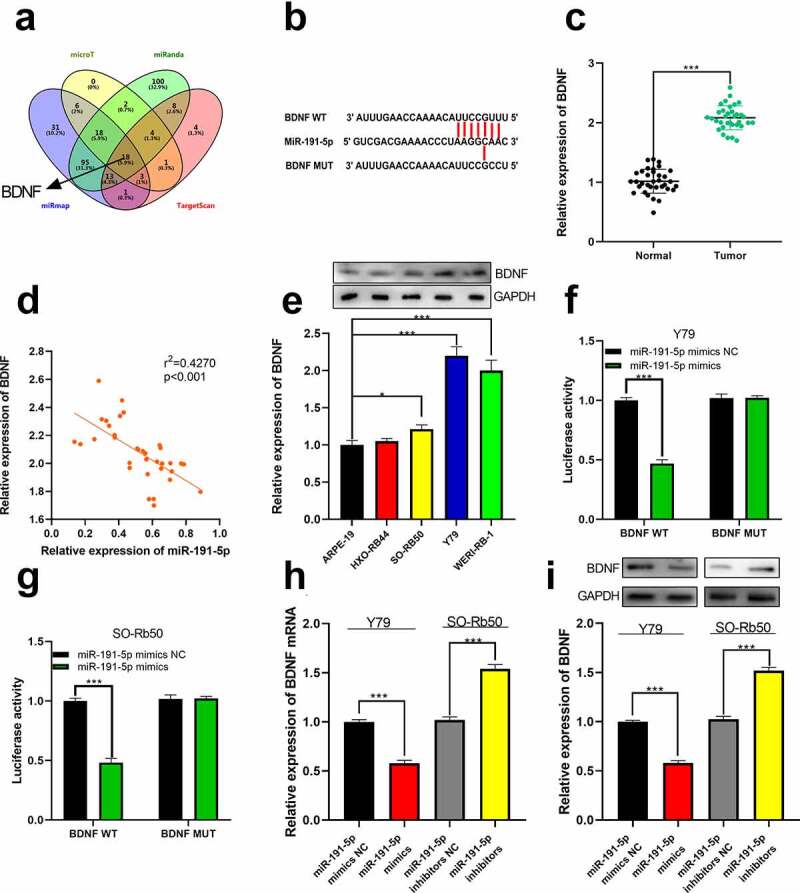
**The relationship between miR-191-5p and BDNF**. (a) The target genes of miR-191-5p were predicted through microT, miRanda, miRmap, and target scan databases. (b) The predicted binding site between miR-191-5p and BDNF 3ʹUTR. (c) The expression of BDNF in normal tissues and RB tissues was detected by qRT-PCR. (d) The expression of BDNF in RB was negatively correlated with miR-191-5p expression in RB samples. (e) The expression of BDNF in RB cell line was detected by Western blot. (f-g) The targeted binding relationship between miR-191-5p and BDNF was verified through a dual-luciferase reporter gene assay. (h) The expression levels of BDNF mRNA in miR-191-5p overexpression and inhibition models were detected by qRT-PCR. (i) Western blot was used to detect the expression level of BDNF in miR-191-5p overexpression and inhibition models

Next, the target genes of miR-191-5p were predicted through microT, miRanda, miRmap, and TargetScan, and as shown, BDNF was one of the potential downstream targets of miR-191-5p (Figure 5(a-b)). We then quantified the expression of BDNF by qRT-PCR. As shown, increased BDNF expression was observed in RB tissues and cell lines, and its expression was negatively correlated with miR-191-5p in clinical samples (Figure 5(c-d)). Furthermore, in RB cell lines, BDNF protein expression was also up-regulated (Figure 5(e)). Dual-luciferase reporter assay manifested that in BDNF WT group, the luciferase activity of reporter vector was remarkably attenuated by miR-191-5p mimics while in BDNF MUT group, that was not affected (Figure 5(f-g)). Further, through qRT-PCR and Western blot, it was demonstrated that miR-191-5p repressed BDNF expression at mRNA and protein levels, unveiling that miR-191-5p could target BDNF (Figure 5(h-i)).

** denotes *p* < 0.01 and *** denotes *p* < 0.001.

### 3.6 The overexpression of miR-191-5p attenuated the effect of XIST

**Figure 6.**
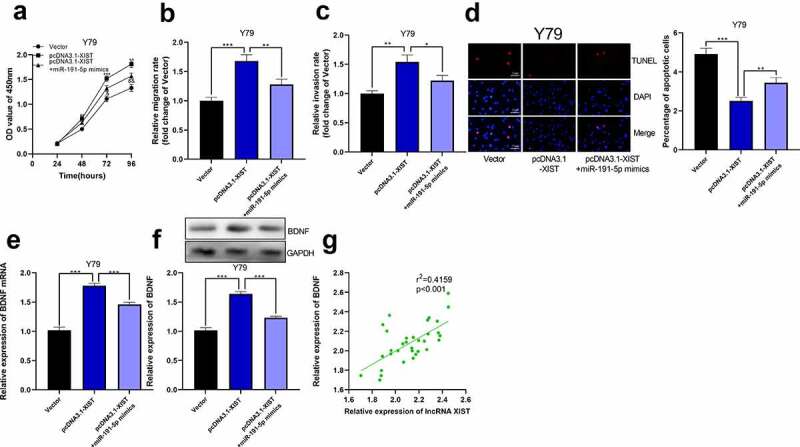
**The overexpression of miR-191-5p reversed the effect of XIST on RB cells**. (a) CCK-8 assay was used to detect the effect of co-transfected miR-191-5p mimics on the proliferation of RB cells with XIST overexpression. (b-c) The effect of co-transfected miR-191-5p mimics on the migration and invasion of RB cells with XIST overexpression was detected by transwell experiment. (d) The effect of co-transfected miR-191-5p mimics on apoptosis of RB cells with XIST overexpression was detected by TUNEL experiment. (e) The expression levels of BDNF mRNA in control, pcDNA3.1-XIST, and pcDNA3.1-XIST+miR-191-5p mimics groups were detected by qRT-PCR. (f) The expression levels of BDNF in control, pcDNA3.1-XIST, and pcDNA3.1-XIST+miR-191-5p mimics groups were detected by Western blot. (g) The expression of BDNF in RB tissue was positively correlated with the expression of XIST

Next, we co-transfected miR-191-5p mimics into Y79 cell line with high expression of XIST. Through CCK-8 experiment, we observed that the proliferation of Y79 cells co-transfected with miR-191-5p mimics and pcDNA3.1-XIST was dramatically lower than that of cells only transfected with pcDNA3.1-XIST (Figure 6(a)). In Transwell experiment, miR-191-5p mimics eliminated the impacts of XIST on promoting the migration and invasion of Y79 cells (Figure 6(b-c)). Through TUNEL experiment, it was elucidated that miR-191-5p mimics counteracted the effect of XIST on inhibiting Y79 cell apoptosis (Figure 6(d)). Furthermore, we observed that overexpression of XIST elevated the expression of BDNF mRNA and its proteins, and miR-191-5p mimics abolished this effect (Figure 6(e-f)). Furthermore, in RB tissues, XIST expression was positively correlated with BDNF expression, which further supported our findings that XIST could indirectly up-regulate BDNF expression (Figure 6(g)).

* denotes *p* < 0.05, ** denotes *p* < 0.01, and *** denotes *p* < 0.001.In A, ** represents the comparison between Vector group and pcDNA3.1-XIST group is *p* < 0.01. *** represents the comparison between Vector group and pcDNA3.1-XIST group is *p* < 0.001.& and && represent that the comparisons between pcDNA3.1-XIST group and pcDNA3.1-XIST+miR-191-5p mimics group are *p* < 0.05 and *p* < 0.01, respectively.

## 4 Discussion

Multiple lncRNAs are reported to partake in the tumorigenesis and development of RB, but still a large number of lncRNAs whose functions in RB remain obscure. XIST mainly plays a role in promoting cancer progression. For instance, in colorectal cancer tissue, XIST expression is remarkably overexpressed, and XIST enhances the proliferation of cancer cells by regulating miR-132-3p/MAPK1 axis [18]. Similarly, in pancreatic cancer, highly-expressed XIST is linked to the unfavorable prognosis of the patients and can facilitate the proliferation of cancer cells by impeding miR-133a expression [19]. Importantly, our research manifested that XIST expression was markedly elevated in RB tissues and cell lines, and remarkably related to RB staging and tumor invasion. Furthermore, through CCK-8, Transwell, and TUNEL assays, it was demonstrated that XIST had the effects of facilitating RB cell proliferation, migration, and invasion, and inhibiting cell apoptosis. Our experimental results suggest that XIST is an oncogenic lncRNA in RB, which is consistent with the previous reports [8,9].

In recent years, the regulatory relationship between lncRNAs and miRNAs has attracted a large amount of attention [20]. Accumulating evidence indicates that, functioning as competitive endogenous RNA (ceRNA) or molecular sponge, lncRNA can bind to specific miRNA and regulate its function, hence affecting cancer procession. In this study, it was confirmed that miR-191-5p was the downstream target of XIST. Moreover, miR-191-5p expression was markedly reduced in RB tissues and cell lines, and miR-191-5p suppressed RB cell proliferation, migration, and invasion, and triggered RB cell apoptosis. In subsequent experiments, we further observed that miR-191-5p partly eliminated the cancer-promoting effect of XIST in RB cells. Consistent with our findings, miR-191-5p in renal cell carcinoma is lowly expressed and exerts cancer-inhibiting effects [14]. Conversely, miR-191-5p is highly expressed and has cancer-promoting effects in some tumors, including osteosarcoma [13]. These studies confirm that miR-191-5p has distinct expression patterns and functions in different cancers. For the first time, our study substantiated that miR-191-5p was a tumor suppressor in RB.

In this study, we authenticated that BDNF was one of the downstream targets of miR-191-5p, and miR-191-5p affected BDNF expression at both mRNA and protein levels in RB cells. Additionally, BDNF was overexpressed in RB tissues and cell lines and its expression was positively regulated by XIST. Since the cancer-promoting effects of BDNF in RB are confirmed by previous studies [16,17], we concluded that XIST exerted its cancer-promoting effect partly by up-regulating the expression of BDNF. BDNF belongs to the neurotrophin family, and its expression is remarkably up-regulated in multiple tumors, and its downstream mechanism is complicated. For instance, in colon cancer, BDNF exerts its cancer-promoting function by facilitating HO-1 expression and VEGF transcription, accompanied by the activation of MAPK signaling pathway [21]. In triple-negative breast cancer, BDNF promotes cancer progression via activating TrkB [22]. Nonetheless, how BDNF affects the tumorigenesis and development of RB requires further exploration in the following studies.

## 5 Conclusion

Collectively, this study substantiates that XIST can indirectly up-regulate BDNF expression by inhibiting miR-191-5p expression to expedite the proliferation, migration, and invasion of RB cells, thereupon promoting RB progression (Figure 7). Our research further explains the impacts of XIST-miR-191-5p-BDNF axis in RB and their mutual regulation mechanism, providing novel insights for the development of novel RB therapeutic drugs.

## Article highlights

·XIST expression was up-regulated in RB tissues

·XIST regulated the proliferation, migration, invasion, and apoptosis of RB cells

·MiR-191-5p was a downstream target of XIST

·BDNF was the downstream target of miR-191-5p, which could be positively regulated by XIST.
